# AKT inhibition interferes with the expression of immune checkpoint proteins and increases NK-induced killing of HL60-AML cells

**DOI:** 10.31744/einstein_journal/2023AO0171

**Published:** 2023-06-06

**Authors:** Sofia Mônaco Gama, Vanessa Araújo Varela, Natalia Mazini Ribeiro, Bruna Bizzarro, Camila Hernandes, Thiago Pinheiro Arrais Aloia, Mariane Tami Amano, Welbert Oliveira Pereira

**Affiliations:** 1 Faculdade Israelita de Ciências da Saúde Albert Einstein Hospital Israelita Albert Einstein São Paulo SP Brazil Faculdade Israelita de Ciências da Saúde Albert Einstein , Hospital Israelita Albert Einstein , São Paulo , SP , Brazil .; 2 Department of Clinical and Experimental Oncology Escola Paulista de Medicina Universidade Federal de São Paulo São Paulo SP Brazil Department of Clinical and Experimental Oncology , Escola Paulista de Medicina , Universidade Federal de São Paulo , São Paulo , SP , Brazil .

**Keywords:** Leukemia, myeloid, acute, AKT, PD-L1, Gal-9, Killer cells, natural

## Abstract

**Objective:**

To determine the role of the AKT pathway in the regulating of natural Killer-induced apoptosis of acute myeloid leukemia cells and to characterize the associated molecular mechanisms.

**Methods:**

BALB/c nude mice were injected with HL60 cells to induce a xenogenic model of subcutaneous leukemic tumors. Mice were treated with perifosine, and their spleens were analyzed using biometry, histopathology, and immunohistochemistry. Gene expression analysis in leukemia cells was performed by real-time PCR. Protein analysis of leukemia and natural Killer cells was performed by flow cytometry. AKT inhibition in HL60 cells, followed by co-culture with natural Killer cells was performed to assess cytotoxicity. Apoptosis rate was quantified using flow cytometry.

**Results:**

Perifosine treatment caused a reduction in leukemic infiltration in the spleens of BALB/c nude mice. *In vitro* , AKT inhibition reduced HL60 resistance to natural Killer-induced apoptosis. AKT inhibition suppressed the immune checkpoint proteins PD-L1, galectin-9, and CD122 in HL60 cells, but did not change the expression of their co-receptors PD1, Tim3, and CD96 on the natural Killer cell surface. In addition, the death receptors DR4, TNFR1, and FAS were overexpressed by AKT inhibition, thus increasing the susceptibility of HL60 cells to the extrinsic pathway of apoptosis.

**Conclusion:**

The AKT pathway is involved in resistance to natural Killer-induced apoptosis in HL60 cells by regulating the expression of immune suppressor receptors. These findings highlight the importance of AKT in contributing to immune evasion mechanisms in acute myeloid leukemia and suggests the potential of AKT inhibition as an adjunct to immunotherapy.

## Highlights

■ AKT signaling sustains the immune evasion mechanisms in acute myeloid leukemia.

■ AKT inhibition increases the susceptibility of acute myeloid leukemia cells to natural Killer-mediated apoptosis.

■ Partial blockage of AKT signaling promotes the suppression of PDL1 and overexpression of pro-apoptotic genes.

**Figure:**
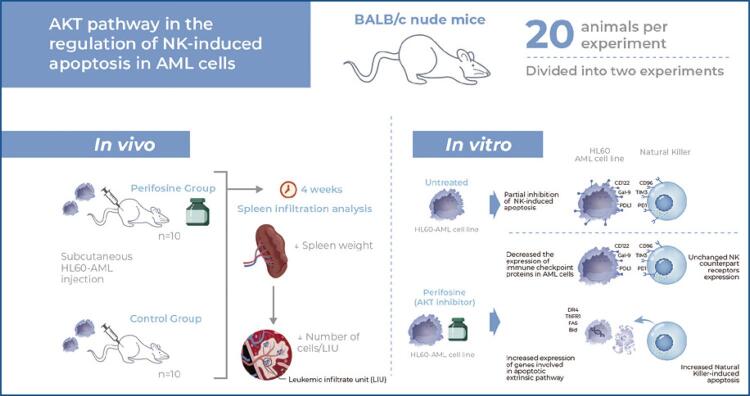

## INTRODUCTION

Acute myeloid leukemia (AML) is a hematological neoplasm characterized by the expansion of malignant myeloid precursors in the bone marrow; it impairs hematopoiesis and evolves into blasts infiltrating the peripheral blood and other tissues. ^( 1 )^ Numerous genetic and immunophenotypic alterations have been described as diagnostic and prognostic markers of AML, but the exact molecular cause of this disease remains to be elucidated. ^( 2 , 3 )^ Next-generation sequencing studies have suggested multiple driver mutations related to AML development. ^( 4 )^ The current standard treatment still comprises chemotherapy regimens ( *e.g.* anthracyclines plus cytarabine), which reduce the bulk of blast cells to undetectable levels. ^( 5 )^ In patients with a poorer prognosis, hematopoietic stem cell transplantation may be indicated, depending on clinical characteristics and treatment outcomes. ^( 6 )^ However, relapse episodes with more resistant leukemic clones are frequent, ^( 7 , 8 )^ with a 5-year survival rate of 28.7%. ^( 1 )^

Alternative treatments, such as tyrosine kinase inhibitors and immunotherapies are promising strategies for improving the prognosis of patients with cancer. ^( 9 , 10 )^ Tyrosine-kinase inhibitors have shown successful outcomes for the treatment of cancers with activated essential pathways, such as breast cancer with EGFR overexpression, BCR-ABL-positive tumors, and myeloproliferative neoplasms carrying the JAK2V617R mutation. ^( 11 )^ Solid tumors have also benefited from the advent of immune checkpoint inhibitors that target programmed cell death protein 1 (PD-1)/ programmed death-ligand 1 (PD-L1). ^( 12 )^ The overexpression of molecules such as PD-L1 and galectin-9 (gal-9) in tumors prevents the activation of cytotoxic immune cells, such as natural Killer (NK) cells, which are vital in suppressing immune evasion of cancers. ^( 12 , 13 )^ These inhibitory monoclonal antibodies are effective in treating a variety of cancers such as advanced melanoma, non-small cell lung cancer, and triple-negative breast cancer. ^( 14 - 16 )^ Nevertheless, immune checkpoint inhibitors have not shown success in AML therapy, due to unknown resistance mechanisms; thus, new combinations with tyrosine kinase inhibitors have been studied in ongoing clinical trials. ^( 17 , 18 )^

Natural Killer cells are essential for the recognition and elimination of neoplastic cells. ^( 19 )^ Inhibitory and stimulatory surface receptors of NK cells can be triggered by tumor proteins and co-receptors, resulting in the inhibition or activation of killing, respectively. ^( 20 )^ Following recognition and activation, NK cells express (and sometimes release) TRAIL and TNF receptor superfamily member 6-ligand (FASL), which bind to death receptor 4 (DR4) and TNF receptor superfamily member 6 (FAS) present on the target membrane, promoting the extrinsic apoptosis pathway. ^( 21 )^ In addition, downstream of the death receptor assembly, the protein BH3 interacting-domain (BID) death agonist amplifies the apoptotic signal by engaging the mitochondrial intrinsic pathway. ^( 22 )^ In contrast, NK cell activity is inhibited following the stimulation of its receptors PD-1, TIM3, and CD96 by their ligands PD-L1, Galectin-9, and CD122/CD155, respectively, contributing to tumor immune evasion. ^( 23 )^

The proliferative and chemotherapy-resistant behavior of AML cells has been extensively investigated and is dependent on intracellular pathways ^( 24 , 25 )^ with important contributions from protein kinase (AKT, also known as PKB), which plays a notable role in the regulation of the cell cycle, survival, and metabolism. ^( 26 )^ Among the different AKT inhibitors, perifosine demonstrated efficacy in targeting the lipid-binding domain of AKT, which is essential for its translocation to the membrane and activation by other kinases, consequently interfering with downstream AKT-mediated phosphorylation. ^( 27 , 28 )^ Perifosine-only regimens failed to confirm the clinical response ^( 29 )^ but the blockage of AKT signaling seems to be effective in reprograming the molecular mechanisms of cancer cells and may act as an adjunct to therapy. ^( 30 , 31 )^

Combined treatments using tyrosine kinase inhibitors and immune therapy approaches have emerged as alternative strategies for leukemia, but the role of AKT in sustaining immune evasion mechanisms is not currently clear. In this study, we investigated the role of AKT in regulating the mechanisms of AML resistance to NK cells.

## OBJECTIVE

To determine the role of the AKT pathway in the regulating of natural Killer-induced apoptosis of acute myeloid leukemia cells and to characterize the associated molecular mechanisms.

## METHODS

### Cell culture and AKT inhibition

The leukemic cell line HL60 was cultured in RPMI 1640 supplemented with Glutamine 2.05mM (Thermo Fisher Scientific, Waltham, MA, USA; CAT 11875-093) and 1% penicillin-streptomycin (Thermo Fisher Scientific; CAT 15140-122) at 37 °C with 5% CO _2_ .

The non-toxic dose and timepoint of treatment of leukemic cells with the AKT inhibitor perifosine (Thermo, CAS Number 157716-52-4) (1μM, 4 hours) were confirmed by vital staining with trypan blue and propidium iodide (PI) (Thermo, CAT BMS500PI) after 18 hours of treatment, and analyzed by Neubauer chamber counting on a microscope and LSR Fortessa FACS (BD Biosciences, San Jose, CA, USA), respectively. For the investigation of AKT phosphorylation status, cells were fixed after 4 hours of perifosine treatment, permeabilized, and stained with anti-AKT-pS473-APC (BD Horizon, CAT560378) and anti-AKT-pT308-APC (BD Horizon, CAT 558275) antibodies according to the BD Phosflow Perm Buffer III protocol (BD Phosflow, CAT 558050). Analyses were performed using an LSR Fortessa FACS (BD Biosciences).

### Real-time PCR

In this study, we used the following TaqMan probes (Thermo Fisher Scientific): GAPDH (Hs03929097_g1), DR4 (Hs00234355_m1), TNR1 (Hs00533568_g1), FAS (Hs00236330_m1), and BID (Hs00609632_m1).

After 18 hours of perifosine treatment *in vitro* , the cells were subjected to Trizol lysis (Thermo Fisher Scientific; CAT 15596018) following the manufacturer’s specifications. cDNA synthesis was performed using Super Script III kit reagents ( Thermo Fisher Scientific; CAT 18080051), and qPCR reactions were performed using TaqMan Universal Master Mix II with UNG kits (Thermo Fisher Scientific; CAT 4440038), according to the manufacturer’s instructions. GAPDH was used as the housekeeping gene, and gene expression was calculated using the formula 2 ^-ΔΔCT^ . The reactions were performed on an ABI Prism 7500 thermocycler (Thermo Fisher Scientific).

### Natural Killer purification

We obtained 50mL of peripheral blood from three healthy donors following ethical approval from the local Ethical Committee *of Hospital Israelita Albert Einstein;* CAAE: 78982417.0.0000.0071; # 2.992.700. Peripheral blood mononuclear cells were separated by density gradient centrifugation of whole blood using a Ficoll-Paque Plus (GE Healthcare, Chicago, IL, USA; CAT GE17-1440-02). Peripheral blood mononuclear cells were labeled with anti-CD56-PC5 antibody (Beckman Coulter, Brea, CA, USA; CAT IM2654U) using 5μL of antibody against 10 ^6^ cells, and NK cells were sorted using a BD FACSAria flow cytometer (>98% purity).

### Surface receptors analysis

The expression of checkpoint receptors in both HL60 and NK cells was assessed using flow cytometry. Cells were cultured in the presence of perifosine for 18 hours and incubated with antibodies for 20 minutes at room temperature, washed twice with 0.5% fetal calf serum supplemented with phosphate-buffered saline (PBS), and analyzed using LSR Fortessa FACS. The antibodies used were anti-CD122-BV421 (BD Horizon, CAT 562887), anti-Galectin-9-PE (BD Pharmingen, CAT 565890), anti-CD274-BB515 (BD Horizon, CAT 564554), anti-CD96-BB515 (BD Horizon, CAT 564774), anti-TIM-3-PE-CF594 (BD Horizon, CAT 565560), and anti-CD279-APC (BD Pharmingen, CAT 558694).

### Cytotoxicity assay

HL60 cells were cultivated in 96-well plates ( $5\times 10^{4}$ cells in 100mL of medium/well) in the presence or absence of 1μM perifosine for 4 hours. Purified NK cells ( $2\times 10^{5}$ ) were added per well to 100mL of the medium, and were co-cultured for 18 hours as follows: HL60 only untreated control (CT), HL60 plus NK (NK), HL60 with perifosine plus NK cells (P+NK), and HL60 with perifosine (P). Cells were stained with PI and analyzed using LSR Fortessa FACS. Natural Killer cells were excluded from the gating strategy based on the FSC and SSC parameters, and the cell death rate of HL60 cells was quantified.

### Leukemic mouse model

Male BALB/c nude mice, 8-10 weeks old (n=20 animals per experiment), were used as the *in vivo* model. A total of 10 ^7^ HL60 cells diluted in 100mL PBS were subcutaneously inoculated into the left flank under isoflurane anesthesia. On day 10, the tumors were visualized, and the mice were randomized into the Control and Perifosine Groups. They received intraperitoneal injections 3 times per week on intercalated days for 3 weeks (the Control Group (n=10) received 100mL of PBS and the Perifosine Group (n=10) received 0.5mg/mL of perifosine diluted in 100mL of PBS). All mice were euthanized at the endpoint (4 weeks after inoculation of leukemic cells). Euthanasia was performed using isoflurane anesthesia, followed by an overdose of xylazine and ketamine anesthesia, carbon dioxide asphyxiation, and cervical dislocation.

Splenic samples were weighed using a high-precision scale and measured using a pachymeter. The volume was calculated according to the following formula, assuming an ellipsoidal geometric shape (V = volume, l = length, h = height, w = width, and π = 3.14).


$$V=\frac{4}{3}\times \pi \times l\times h\times w$$

All experimental procedures were performed in triplicates in a mouse care laboratory.

### Histology and immunohistochemistry

The spleens were cut into symmetrical fragments and fixed in 4% paraformaldehyde for subsequent tissue assembly in paraffin blocks.

Histology labels were processed with hematoxylin (Merck, 517-28-2) and eosin (Merck, 239-138-3) to visualize tissue architecture and cell distribution. Immunohistochemistry with anti-human CD117 (Dako, Santa Clara, CA, USA; CAT 10124187) was used to identify human HL60 cells in the murine spleen. After staining, the slides were analyzed using an optical microscope (Olympus IX51). CD117-positive cells (HL60) and leukemic infiltrate colonies were counted from 5 representative **×** 50 fields using Zen 2.6 blue edition image viewing software.

### Statistical analysis

Statistical analyses were performed using Prism 8 (GraphPad Software, La Jolla, CA, USA). The Mann-Whitney test was used to compare the two independent groups. One-way analysis of variance followed by Tukey’s multiple comparisons test was used to analyze three or more variables, assuming parametric data. Statistical significance was set at p<0.05, and the variations in data were evaluated as the mean ± standard error. For the leukemic infiltrate unit study, the median of each field was calculated using an independent *t* -test. All experiments were performed in triplicate.

## RESULTS

**Figure 1:**
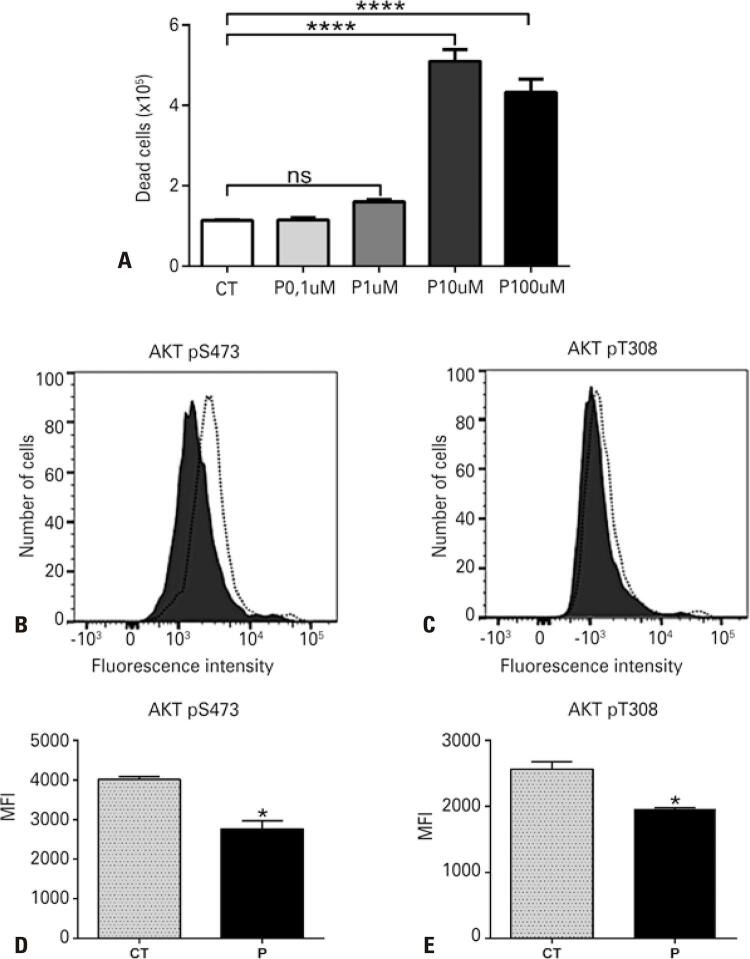
Perifosine treatment shows inhibition of S473 and T308 AKT phosphorylation. (A) HL60 cells were cultivated without (control - CT) or with perifosine (P) under different concentrations for 18 hours and cell death was assessed using trypan blue staining. (B, C, D, and E) The HL60 cells were treated with 1µM perifosine for 4 hours and AKT activation was evaluated by Phosflow staining. Histograms demonstrate decreased fluorescence of AKT (B) pS473 and (C) pT308 after inhibition with perifosine. Dotted line: untreated control; filled histogram: perifosine-treated cells. Bar graphs show inhibition of AKT (D) pS473 and (E) pT308 activation after pharmacological intervention with perifosine. Analysis of variance with the Bonferroni post hoc test was applied * p<0.05; **** p<0.0001. ns: non-significant; CT: control; P: perifosine; MFI: mean fluorescence intensity.

The sublethal dose of perifosine for HL60 cells was first confirmed ( Figure 1A ) and its effect on AKT inhibition was determined by evaluating the phosphorylation of serine 473 ( Figure 1B and D ) and threonine 308 ( Figure 1C and E ) after 4 hours of treatment. Since the reduction was observed in both phosphorylation sites, we considered 1µM of perifosine as a sufficient dose to partially impair the downstream pathway without causing cell death ( Figure 1 ).

**Figure 2:**
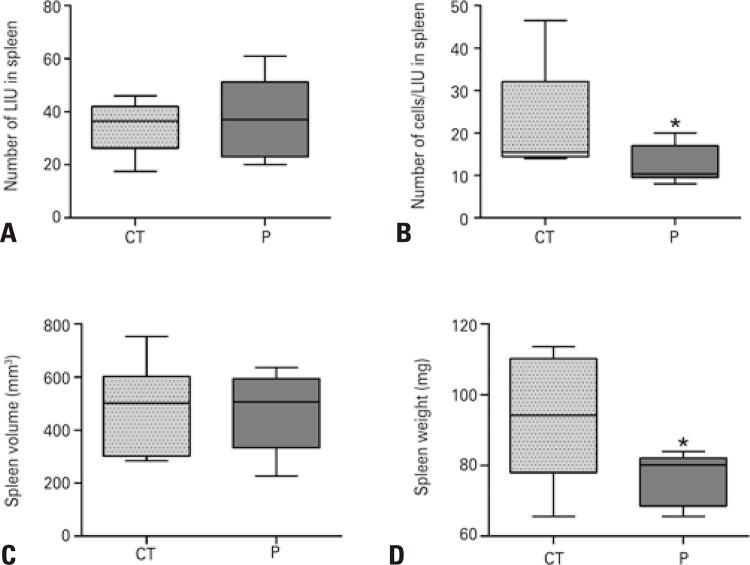
Perifosine treatment diminishes the cellularity of leukemic infiltrate units in the spleen of BALB/c nude mice. (A) Data demonstrate a similar number of leukemia infiltrate units in both groups; (B) The Perifosine Group has a diminished number of total cells (HL60) per leukemia infiltrate units than the Control Group; (C) Inhibition of AKT results in similar splenic volume in both groups; (D) Inhibition of AKT leads to decreased splenic weight when compared to the Control Group. Each experiment was performed in triplicates * p<0.05. CT: control, P: perifosine; LIU: leukemic infiltrate units.

The effects of AKT inhibition on HL60 survival were investigated using an *in viv* o model generated by subcutaneous injection of HL60 cells into the flank of BALB/c nude mice. Spleen infiltration is a common histological marker of AML, and was thus investigated in this model. Although the histopathological analysis did not reveal any difference in the tissue architecture between untreated and perifosine-treated mice, we observed leukocyte infiltration in the spleen of both groups, with massive leukemic infiltration units (LIU) containing human-CD117 ^+^ cells ( Supplementary 1 ). Microscopic analysis revealed that perifosine treatment had no effect on the number of LIU per spleen, but reduced the number of leukemia cells in each LIU, which correlates with decreased spleen weight in treated animals ( Figure 2 ). These results suggest that AKT inhibition impairs the survival and infiltration of leukemia cells.

**Supplementary 1:**
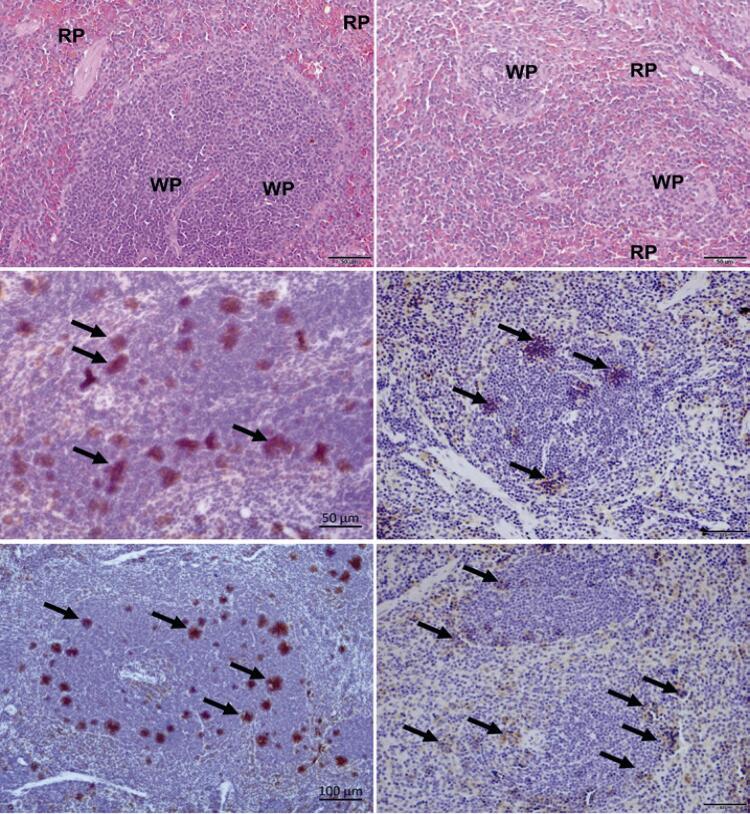
Histopathological analysis of HL60 infiltration in the spleen. (A) Histological photomicrographs of the spleen in perifosine-untreated Control Group; (B) Perifosine-treated group. Immunohistochemistry (anti-human CD117+ cells) photomicrographs reveal; (C) Leukemic infiltrate units in the spleen of the Control Group; (D) Leukemic infiltration units in the spleen of the treatment group; (E) Leukemic infiltrate units in the spleen of the Control Group and (F) Leukemic infiltration units in the spleen of the treatment group. The arrows in the figures indicate the leukemic infiltrate units expressing human CD117 RP: red pulp; WP: white pulp.

**Figure 3:**
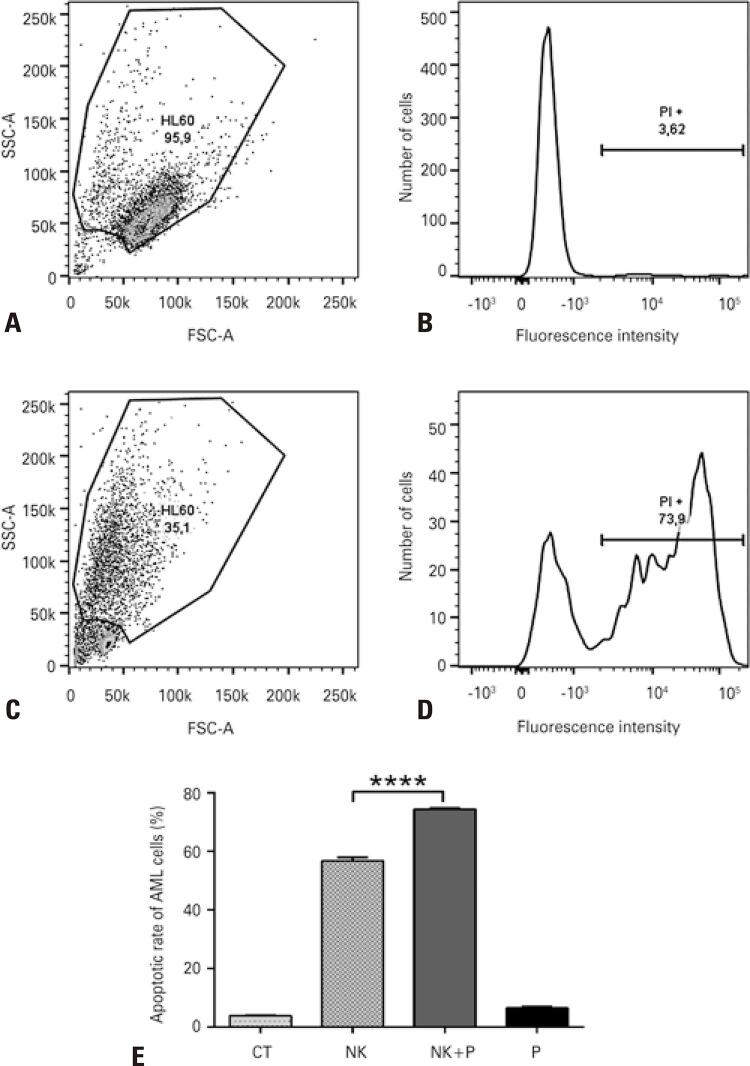
AKT inhibition increases leukemic cell susceptibility to apoptosis induced by NK cells. (A) Gating strategy to exclude dead cells, debris, and NK cells in control (upper) and NK+perifosine (lower) groups based on standard size and granularity; (B) PI-positive cells indicate the leukemic apoptotic rate after co-culture with NK cells in control (upper) and NK+perifosine (lower) groups; (C) Gating strategy to exclude dead cells, debris, and NK cells in control (upper) and NK+perifosine (lower) groups based on standard size and granularity; (D) PI-positive cells indicate the leukemic apoptotic rate after co-culture with NK cells in control (upper) and NK+perifosine (lower) groups; (E) Cell death rate in the control (CT) group, which includes untreated HL60; NK cells in co-culture with HL60 (NK); NK cells in co-culture with HL60 plus perifosine (NK+perifosine); and HL60 treated with perifosine (P). This graph demonstrates a significant increase in the rate of NK cells-induced HL60 apoptosis in the presence of perifosine (1μM, 4 hours). Each experiment was performed in triplicates using NK cells derived from different donors per experiment ****p <0.0001.

NK cells are the only functional anti-tumor immune compartments in BALB/c nude mice, and AKT inhibition could directly impact the performance of these cells in HL60 infiltration and survival. We next investigated the ability of NK cells to induce apoptosis in HL60 cells under perifosine treatment. AKT inhibition substantially increased NK-mediated HL60 killing after 18 h of co-culture ( Figure 3 ). Without treatment, NK cells were able to induce AML cell death of approximately 55%, but this performance was amplified to 75% when perifosine was added to the culture. These results demonstrated that the AKT pathway is sustained, at least partially, by the mechanisms of resistance to NK-induced apoptosis.

**Figure 4:**
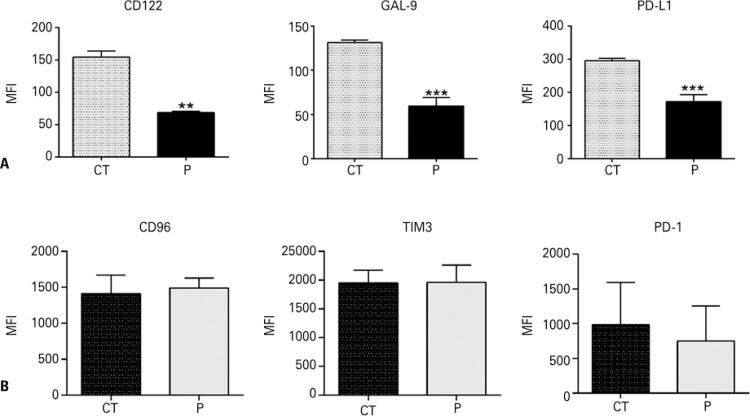
AKT inhibition decreases the expression of immune checkpoint markers and does not affect the expression of their receptors on NK cells. (A) Perifosine decreases the expression of immune suppressor molecules CD122, Gal9, and PDL1 in AML cells; (B) Perifosine does not interfere with the expression of NK receptors, such as CD96, TIM3, and PD1. The cells were treated with perifosine for 18 hours and surface protein expression was assessed by flow cytometry. Each experiment was performed in triplicates using NK cells derived from different donors per experiment **p<0.001. CT: control; MFI: mean fluorescence intensity; P: perifosine.

Tumor cells have been shown to escape killing by expressing surface immune suppressor molecules, and CD122, Galectin-9, and PD-L1 are commonly used to impair NK and T-lymphocyte-mediated attacks. Since perifosine improved NK-induced apoptosis in HL60 cells, we next investigated the ability of the AKT pathway to sustain this mechanism. We observed an exuberant expression of CD122, Galectin-9, and PD-L1 in HL60 cells ( Figure 4A , gray bars), and found that AKT inhibition primarily decreased the surface expression of these immune checkpoint molecules in leukemia cells ( Figure 4A , black bars). The expression of their corresponding receptors, CD96, TIM3, and PD1, in the NK cell plasma membrane, was exuberant and unchanged by perifosine treatment ( Figure 4B ). These results show that the AKT pathway is important for sustaining the expression of immune suppressor molecules in HL60 cells, but is not related to the expression of the co-receptors in NK cells.

**Figure 5:**
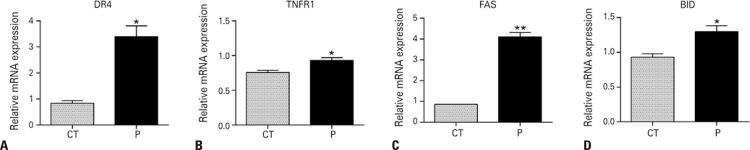
AKT inhibition increases the expression of genes involved in the apoptosis extrinsic pathway in HL60. Perifosine elevated the expression of death receptors DR4, FAS, and TNFR1, as well as of BID, a pro-apoptotic gene responsible for converting the extrinsic into the intrinsic apoptotic pathway. The cells were treated with perifosine for 18 hours and gene expression was assessed by real-time PCR. The experiment was performed in triplicates * p <0.05; **p <0.01. CT: control; P: perifosine.

The increase in susceptibility to NK cells observed during perifosine treatment could also be related to a differential apoptotic gene expression profile in HL60 cells. An important NK-inducing apoptosis mechanism involves death receptors, and tumor cells may be more susceptible or resistant to this process, depending on the expression of extrinsic pathway genes. We found overexpression of death receptor 4 (DR4), FAS (also known as TNFRSF6 - tumor necrosis factor receptor superfamily member 6), and tumor necrosis factor receptor 1 (TNFR1) after AKT inhibition. BH3 interacting-domain, a pro-apoptotic member of the Bcl-2 family responsible for amplifying the intracellular apoptotic signaling downstream of extrinsic pathway activation, was also overexpressed after perifosine exposure ( Figure 5 ). This gene profile corroborates that HL60 is more sensitive to NK cell attack, potentially via a TRAIL/FASL/ TNF-mediated mechanism of apoptosis.

## DISCUSSION

The relevance of the AKT pathway in hematological malignancies has been associated for decades with the regulation of the cell cycle, proliferation, apoptosis, and metabolism of cancer cells. ^( 32 )^ In a preclinical model, PI3k-AKT inhibition induced apoptosis in AML cell lines and primary human AML cells in a dose-dependent manner. ^( 33 )^ In the present study, we performed *in vitro* experiments using a sub-lethal dose of HL60 to circumvent the direct apoptotic effect on the cells. This allowed us to verify the efficacy of partial inhibition of AKT in sensitizing HL60 cells to NK-induced apoptosis. Our data revealed that AKT sustains the expression of PD-L1 and other immune suppressor receptors in HL60 cells, and its inhibition not only suppresses these proteins but also induces overexpression of pro-apoptotic genes associated with NK-induced apoptotic pathways. We found an improvement in killing by NK against AML cells, which strongly suggests that even partial AKT inhibition could contribute to NK-mediated elimination of leukemia cells, potentially in combination with immunotherapies. Co-treatment using the blockage of the AKT pathway has already been tested in leukemia and other cancers. ^( 34 - 36 )^ A phase I study with multiple myeloma patients showed that the efficacy of perifosine plus lenalidomide-dexamethasone can be favorable, especially for patients with an increased basal rate of p-AKT. ^( 37 )^ However, as demonstrated in a phase II clinical trial, AKT inhibition as a single approach had insufficient activity in relapsed/refractory AML patients. ^( 38 )^ Another phase I study revealed that co-treatment with perifosine and UCN-01 (a PDK1 inhibitor) regimens was not effective in relapsed and refractory acute leukemia patients. ^( 29 )^ Probably the impact of AKT inhibition depends on the molecular context of the tumor, such as cancer type, gene expression profile, tumor burden mutation, and drug combination.

Immunotherapies have profitably evolved in the last decade, however, their success largely depends on the proper function and regulation of the patient’s immune system. ^( 39 )^ Ongoing clinical trials of PD-1 inhibitors, such as Nivolumab, Pembrolizumab, Durvalumab, and Atezolizumab, show promising results in AML treatment. ^( 40 )^ At the same time, immune evasion mechanisms hinder these innovative approaches. ^( 41 )^ Malignant cells expressing PD-L1 were shown to release PD-L1-containing exosomes, which work as an immune suppressor barrier in the tumor microenvironment. ^( 42 )^ These exosomes may also compete with checkpoint inhibitors to preserve target cells. Acute myeloid leukemia cells express PD-L1 and other immunosuppressive ligands ^( 43 , 44 )^ however, it is still unclear whether leukemia cells might have additional effects on PD-L1/PD-1 interaction besides inhibition of T and NK cells. In breast cancer cell lines, the interaction of PD-L1 with PD-1 increases PI3K/AKT signaling, which contributes to the resistance of neoplasms to anti-tumor agents. ^( 45 )^ In diffuse large B-cell lymphoma, the concomitant expression of PD-L1 and p-AKT is related to unfavorable prognosis, and a report indicates that AKT/mTOR signaling could also be stimulated by PD-1/PD-L1 interaction. ^( 46 )^ Here, we demonstrated that AKT inhibition decreases the protein expression of Gal-9, CD122, and PD-L1. The impact of this downregulation is not restricted to the decreased capability of AML cells to avoid NK-induced apoptosis. The diminished expression of these ligands due to AKT inhibition could potentially mitigate other intracellular leukemogenic mechanisms. In light of this scenario, PD-1/PD-L1 and AKT co-inhibition could be considered as a novel therapeutic approach for patients who do not respond to checkpoint inhibitors. Furthermore, the poor effect of anti-PD-1/PD-L1 immunotherapy in some patients with leukemia could be explained by the neglected expression of Gal-9 and CD122, which would reveal the possibility of new molecular targets for checkpoint immunotherapies.

NOD-scid-gamma mice are commonly used as xenograft models to reproduce a leukemia-like disease, ^( 47 )^ in which an important blast infiltration in the spleen and a preferentially located HL60 infiltration in the bone marrow are observed. ^( 48 )^ These animals are deficient in NK cells, ^( 49 )^ therefore, to investigate the potential effect of AKT inhibition in improving NK cell effectiveness *in vivo* , we decided to use BALB/c nude mice, another well-known model with NK cells as the main anti-tumor immune component. ^( 50 )^ We found similar LIU in the spleen of all animals; however, perifosine-treated animals presented a decreased number of AML cells per LIU, which corroborated with the results of decreased spleen weight and indicated that AKT inhibition may not interfere with cellular mechanisms related to infiltration, such as migration and adhesion. Instead, we suggest that HL60 has an impaired ability to escape from NK recognition, favoring killing by NK cells in the treated mice group, potentially through the mechanisms demonstrated *in vitro* .

In recent years, preliminary results of clinical trials on high-dose NK cell allogeneic therapy have shown a good safety profile in the improvement of the anti-leukemic activity. ^( 51 , 52 )^ Furthermore, genetically engineered modified chimeric antigen receptor (CAR) -NK cells are being developed against AML and could be a potent strategy for targeting AML cells and inducing NK cell-mediated killing, including death through the secretion of FAS-L and TRAIL. ^( 53 , 54 )^ According to our results, AKT inhibition increased the sensitivity of AML cells to NK-induced apoptosis. In this regard, NK-based cell therapy strategies may be beneficial for AML treatment of non-responding patients when combined with perifosine or other AKT inhibitors.

## CONCLUSION

This study reinforces the relevance of AKT activation/inhibition in acute myeloid leukemia. This pathway is important for immune evasion because it sustains the expression of PD-L1, Gal-9, and CD122, which may suppress the action of natural Killer cells and T lymphocytes. Moreover, the anti-apoptotic role of AKT assists leukemia cells by diminishing the extrinsic pathway machinery involved in TRAIL- and FASL-induced apoptosis. AKT inhibition is a successful strategy for reversing evasion mechanisms of cellular cancer and contributing to the elimination of leukemia cells via an natural Killer-mediated mechanism, which strongly advocates AKT as a molecular target in current and future combined treatments to improve immunotherapy outcomes.
